# Vitamin D Receptor Gene Polymorphisms Affect Osteoporosis-Related Traits and Response to Antiresorptive Therapy

**DOI:** 10.3390/genes14010193

**Published:** 2023-01-11

**Authors:** Vladimira Mondockova, Veronika Kovacova, Nina Zemanova, Martina Babikova, Monika Martiniakova, Drahomir Galbavy, Radoslav Omelka

**Affiliations:** 1 Department of Botany and Genetics, Constantine the Philosopher University in Nitra, 949 01 Nitra, Slovakia; 2 Department of Zoology and Anthropology, Constantine the Philosopher University in Nitra, 949 01 Nitra, Slovakia; 3 Private Orthopedic Ambulance, 949 01 Nitra, Slovakia

**Keywords:** osteoporosis, *VDR* gene polymorphism, bone mineral density, fracture, ibandronate, raloxifene, bone turnover markers, pharmacogenetics, treatment efficacy

## Abstract

The present study analyzed the effect of vitamin D receptor (*VDR*) gene polymorphisms (ApaI, TaqI, BsmI, FokI, and Cdx2) on bone mineral density (BMD), biochemical parameters and bone turnover markers, fracture prevalence, and response to three types of antiresorptive therapy (estrogen-progesterone, raloxifene, and ibandronate) in 356 postmenopausal women from Slovakia. Association analysis revealed a significant effect of BsmI polymorphism on lumbar spine BMD, serum osteocalcin (OC), and β-CrossLaps levels. While ApaI and Cdx2 polymorphisms were associated with OC and alkaline phosphatase, TaqI polymorphism affected all turnover markers. ApaI, TaqI, and BsmI genotypes increased the risk of spinal, radial, or total fractures with odds ratios ranging from 2.03 to 3.17. Each of therapy types evaluated had a beneficial effect on all osteoporosis-related traits; however, the *VDR* gene affected only ibandronate and raloxifene treatment. ApaI/aa, TaqI/TT, and BsmI/bb genotypes showed a weaker or no response to ibandronate therapy in femoral and spinal BMD. The impact of aforementioned polymorphisms on turnover markers was also genotype dependent. On the contrary, only TaqI and BsmI polymorphisms influenced raloxifene therapy, even only in lumbar spine BMD. These results point to the potential of using the *VDR* gene in personalized pharmacotherapy of osteoporosis.

## 1. Introduction

Osteoporosis (OP) is a metabolic skeletal disorder prevalent worldwide, characterized by low bone mineral density (BMD), degenerative micro-architectural deterioration of bone tissue with a potential high risk of hip, spinal, and wrist fractures [1]. Due to the aging of the population, the annual number of osteoporotic fractures is expected to increase by 25% in the next 10 years in Europe [2]. In Slovakia, annual deaths due to fractures are nowadays the highest in Europe [3]. 

OP is a disease with a polygenic type of inheritance. Many factors, including age, gender, physical activity, or menopausal status, affect the risk of this disease. The importance of genetic factors in the pathophysiology of OP reflects a high degree of heritability of individual parameters involved in bone strength [4]. Therefore, a lot of effort is devoted to their detection and testing the influence of associated genes in different populations. 

However, the heritability of bone loss and fractures decreases with increasing age and lifestyle factors become more important [5]. It is difficult to determine the extent to which lifestyle effects are influenced by diet alone, due to difficulties in quantifying environmental exposures, both current and lifetime. In addition, the impact of diet on bone health may be more complex and may depend on an individual’s genotype, gene–diet interactions, or gut microbiome composition and function [6,7]. 

A balanced diet that meets the daily caloric needs and contains the required daily intake of calcium (Ca) and vitamin D is a key factor in achieving maximal bone mass as well as reducing the rate of bone loss in the elderly. In addition, other vitamins, minerals, macronutrients, and polyphenols are also involved in bone metabolism and appear to be promising for the prevention and treatment of osteoporosis [6,8,9]. 

Vitamin D is an important vitamin and steroid pro-hormone that plays a critical role in bone mineralization and metabolism. It regulates the concentrations of serum calcium (sCa) and serum phosphate (sP), ensures adequate deposition of hydroxyapatite in the bone matrix, and affects skeletal development, maintenance of skeletal architecture, hormone secretion, and immune function [10]. This action of vitamin D is mediated through the vitamin D receptor (VDR) that specifically binds to 1.25 dihydroxyvitamin D_3_. Genetic revelations have started explaining the complex associations of vitamin D signaling and bone health [11,12]. For this reason, the gene encoding VDR is considered an important candidate for the genetic regulation of bone strength, bone homeostasis, and metabolism. The approximately 75 kb gene is located on chromosome 12q13.11, containing 12 exons and nearly 300 different polymorphisms [13,14]. Variability in the *VDR* gene has been identified as a possible contributor to OP. Five polymorphisms of the *VDR* gene are most widely studied in association with BMD, bone turnover markers, or fracture risk: ApaI (rs7975232 A/C), BsmI (rs1544410 A/G), FokI (rs2228570 T/C), TaqI (rs731236, T/C), and Cdx2 (rs11568820 G/A) [15,16]. Although several studies investigating the relationship between OP and *VDR* gene polymorphisms have been published, the results are often ambiguous [17,18,19]. 

Genetic variability in the *VDR* gene may also have important pharmacogenetic applications. It is known that individual variants in genes not involved in drug metabolism may also influence the risk for variable drug responses and could be of a great importance for clinical applications [20]. In general, OP therapies fall into two classes, antiresorptive drugs (e.g., estrogen, selective estrogen receptor modulators, bisphosphonates, and calcitonin), decreasing the rate of bone resorption, and anabolic drugs (e.g., parathyroid hormone and related peptide analogs, and sclerostin inhibitors), stimulating bone formation [21,22]. Bisphosphonates (BPs; alendronate, risendronate, ibandronate, or zoledronic acid) represent the first-line therapy for the prevention of osteoporotic fractures [23]. Bone resorption is suppressed by binding to hydroxyapatite, and BPs are absorbed by bone and block osteoclast activity [24]. Since postmenopausal estrogen deficiency is the main cause of bone loss in OP, hormone therapy (HT) has been suggested for its prevention [25,26]. HT in the form of either combined estrogen and progesterone or estrogen alone has been shown to be effective in reducing vertebral and non-vertebral fractures in postmenopausal women [27]. Over time, the use of HT has declined considerably due to safety concerns. On the other hand, selective estrogen receptor modulators (SERM) are non-steroidal synthetic drugs with similar effects on bone as estrogen, but did not influence adversely the breast and endometrium. The most frequently used SERM in postmenopausal osteoporotic women is raloxifene [28,29]. 

The present study analyzed the effect of five *VDR* gene polymorphisms (ApaI, TaqI, BsmI, FokI, and Cdx2) on BMD, bone turnover markers, biochemical parameters, prevalence of fractures, and response to three types of antiresorptive therapy in postmenopausal women from southern Slovakia. This region is considered a historically significant Hungarian-Slavic contact zone. We have previously demonstrated that the estrogen receptor 1 gene can contribute to changes in BMD unrelated to fracture prevalence and also influences the response to hormonal and raloxifene therapy in this population [30].

## 2. Materials and Methods

### 2.1. Studied Population

The present study included 356 postmenopausal women from southern Slovakia aged from 45 to 85 years (63.13 ± 0.45 years) who were followed up as part of the basic diagnostic screening for OP. Strict inclusion criteria were used for participant’s selection. Women who had serious endocrine or chronic diseases, obese women (BMI = 30.0 kg/m^2^ and above), and individuals with premature menopause, premature ovarian insufficiency, or serious menstrual cycle disturbances were excluded from the study. None of the subjects had taken any medication related to affect bone metabolism (glucocorticoids, hormones, and previous antiosteoporosis drugs). In some cases, not all genotypes were available for all samples, so finally 338, 329, 292, 316, and 342 samples were evaluated for ApaI, BsmI, FokI, TaqI, and Cdx2 polymorphisms, respectively. The study group included 214 postmenopausal women with diagnosed OP (60.11%), the remaining women were considered controls in the statistical models. All women with diagnosed OP were subsequently included in the pharmacogenetic study.

### 2.2. Clinical Data Acquisition

Information about medical history, age, and lifestyle habits were assessed using a questionnaire accomplished by the respondents and reviewed by a qualified physician. Body mass index (BMI) was computed as weight in kilograms divided by height in meters squared. Presence (P) or absence (A) of total, femoral, radial, and spinal fractures (also including compression fractures) were diagnosed by clinical evaluation based on X-rays radiographs. BMD was determined by dual energy X-ray absorptiometry (Hologic Discovery DXA, Canada). Femoral (FBMD) and lumbar spine vertebrae BMD (LBMD) was measured at the femoral neck and at the lumbar spine (L2–L4) and expressed by the t-score. The same densitometer was used for all tested women. Biochemical markers included bone isoenzyme of alkaline phosphatase (bALP), osteocalcin (OC), and β-CrossLaps (β-CTx). bALP (μkat/L) was determined by immunoenzymatic assay (Beckman Coulter Access Ostase assay, Beckman Coulter, Brea, CA, USA). OC (μg/L) and β-CTx (ng/L) were measured by electrochemiluminescence immunoassay with cobas e411 (Roche Diagnostics, Basel, Switzerland). Concentrations of sCa (mmol/L) and sP (mmol/L) were analyzed by photometric assay with cobas c311 (Roche Diagnostics, Basel, Switzerland). All measurements were performed by accredited clinical laboratories in Nitra (Slovakia).

### 2.3. Genetic Analysis of the VDR Gene Polymorphisms

Genomic DNA was extracted from EDTA blood samples using the commercial blood isolation kit (SiMax™ Genomic DNA Extraction Kit, Beijing, China). *VDR* polymorphisms were determined by PCR-RFLP using primers published by Vandevyver et al., Flügge et al., and Harris et al. [31,32,33]. PCR was performed as follows: 95 °C for 5 min followed by (Apa, Taq, BsmI) 94 °C for 30 s, 64 °C for 40 s, and 72 °C for 1 min. The reaction consisted of 35 cycles, and it was completed by a final extension cycle at 72 °C for 7 min. The PCR cycle conditions for FokI and Cdx2 polymorphisms included denaturation at 94 °C for 5 min, followed by 35 cycles at 94 °C for 30 s, 61 °C for 40 s, and 72 °C for 30 s, and one final cycle of extension at 72 °C for 7 min. We used 100–200 ng of genomic DNA, 0.5 µmol/L of forward and reverse primers, 1xTaq polymerase buffer (1.5 mmol/L MgCl_2_), dNTPs (2.0 mmol/L each), and 1.0 unit of Taq DNA polymerase (BioTherm Taq DNA Polymerase, Genecraft, Jakarta Barat, Indonesia). After amplification, the PCR product was digested with appropriate restriction endonucleases (Thermo Scientific™, Waltham, MA, USA) at 37 °C overnight and separated by electrophoresis in agarose gel containing ethidium bromide (Table 1). The gels were documented by DNR Bio-Imaging Systems (MiniBIS Pro, Jerusalem, Israel). The commonly used nomenclature indicating presence of the restriction site in small letters and its absence with capitals is displayed in Table 1.

### 2.4. Pharmacogenetic Study

Data from osteoporotic women (N = 214) receiving regular antiosteoporotic therapy for 4 years were analyzed. BMDs (FBMD and LBMD) and bone turnover markers were measured at study entry and after the treatment. The therapy types included application of estrogen-progesterone hormone therapy (EPT; 17β-estradiol in combination with progesterone, 1 mg/day for both; N = 76), SERMs/raloxifene (60 mg/day; N = 64), or BPs/ibandronate (150 mg/month; N = 74). During the treatment, all participants received Ca (1000 mg/day) and vitamin D (800 IU/day) supplementation.

### 2.5. Statistical Analysis

Statistical analysis was realized using IBM SPSS Statistics 28.0 software (IBM, New York, NY, USA). The data were summarized as mean ± SE (Standard Error of the Mean) for quantitative variables and as frequencies for qualitative variables. Genotype distribution was tested for Hardy–Weinberg equilibrium using the chi-square test. Analysis of covariance (General Linear Model procedure, GLM) was performed for quantitative variables assessment among the genotypes after correction of the measurements for age and BMI. The normality of data was evaluated using the Shapiro-Wilk test. Possible interactions (significance interval) were verified using Johnson-Neyman procedure [34]. For evaluation of fracture incidence Binary Logistic Regression with the genotype, age and BMI as covariates was used to estimate the odds ratio (OR) and 95% confidence interval (CI) for the association between fracture risk and *VDR* genotypes. The effect of genotypes on BMD change during a treatment was evaluated by variance analysis for repeated measurements using GLM, where the measured trait before and after treatment represented a repeat dependent variable and the individual genotypes were fixed effects. Corrections for multiple testing of genotype effects were performed by Bonferroni correction. According to the relatively small sample size, we calculated the ideal statistical power based on the observed effect size. Observed power was determined over 80% with small to medium effect size. The p-value less than 0.05 was considered statistically significant.

## 3. Results

### 3.1. Frequencies of VDR Genotypes and Alleles

Frequencies of genotypes and alleles for *VDR* gene polymorphisms in the total analyzed population are shown in Table 2. The distribution of genotypes agreed with that expected according to the Hardy–Weinberg equilibrium. The highest frequencies of heterozygous genotypes for all polymorphisms examined were recorded. Clinical characteristics and parameters of the study population are shown in Table 3.

### 3.2. Associations between VDR Gene Variants and Osteoporosis-Related Traits

Associations of ApaI, TaqI, BsmI, FokI, and Cdx2 genotypes with the osteoporosis-related characteristics are presented in Table 4. 

The results for *VDR*/ApaI polymorphism showed that serum OC level was significantly higher in women with aa genotype in comparison with heterozygous Aa genotype (*p* = 0.001). There were no significant differences in the remaining analyzed traits (FBMD, LBMD, bALP, β-CTx, sCa, and sP). 

Genotypes of TaqI polymorphism were associated with bone turnover markers but not with femoral and spinal BMD. Individuals with TT genotype had significantly elevated levels of all bone turnover markers (bALP, OC, β-CTx) in comparison with Tt genotypes (*p* < 0.05). Significantly increased β-CTx levels were also found in tt genotype carriers (*p* < 0.05). sCa and sP levels were not associated with TaqI genotypes. 

We recorded a significant impact of the *VDR*/BsmI polymorphism on LBMD. Women with homozygous bb genotype had lower BMD values compared to Bb genotype (*p* < 0.05). Similarly, biochemical markers of bone turnover were also associated with BsmI polymorphism. Concentrations of OC and β-CTx were significantly elevated in bb genotype carriers (*p* < 0.05). On the other hand, women with bb genotype had reduced sP levels compared to the other genotypes (*p* < 0.05). 

No differences were determined between the *VDR*/Cdx2 genotypes and BMD at any measured site. A significant effect of Cdx2 genotypes was recorded only in relation to bALP. Subjects with GG genotype had significantly higher bALP levels (*p* < 0.05) compared to those with AA genotype. 

Finally, no association of *VDR*/FokI genotypes with any of the analyzed parameters was found.

### 3.3. Associations between VDR Gene Variants and Fracture Incidence

Binary logistic regression analysis revealed that *VDR* genotypes were significantly associated with clinical fracture prevalence after adjustment for age and BMI (Table 5). 

ApaI genotypes affected the incidence of spinal, radial as well as total fractures. The estimated odds ratio for genotype showed that women with aa genotype had a higher chance of spinal (OR = 2.15; *p* = 0.027), radial (OR = 3.173; *p* = 0.003), as well as total fractures (OR = 2.434; *p* = 0.007) versus Aa genotype. 

On the other hand, TaqI polymorphism exclusively affected only spinal fracture incidence. Participants with homozygous TT had a higher chance of spinal fracture compared to heterozygous Tt genotype (OR = 2.386; *p* = 0.01). 

BsmI polymorphism was positively associated with spinal and total fracture prevalence. For spinal fractures, OR was 2.48 (*p* = 0.005) for bb genotype compared to Bb genotype. The calculation for total fractures revealed OR = 2.034 (*p* = 0.018), where women with genotype bb were at higher risk. 

None of the other analyzed polymorphisms (Cdx2, FokI) of the *VDR* gene influenced fracture prevalence. Femoral fractures were not included in the analysis because of a small number of femoral fracture carriers (N = 4).

### 3.4. Associations between VDR Gene Variants and Therapy Response

Pharmacogenetic analysis showed that therapy types evaluated positively affected all monitored osteoporosis-related traits after 48 months of treatment (*p* = 0.001) (Table 6). Most patients treated with analyzed antiresorptive drugs had significantly higher t-scores and decreased turnover marker levels after therapy (Supplementary file/Figures S1–S3). However, when considering the effects of the *VDR* gene, significant differences in the treatment efficiency between *VDR* genotypes were revealed only in ibandronate- and raloxifene-treated groups (Table 7 and Table 8). No significant differences in BMD or bone turnover markers were identified between *VDR* genotypes after estrogen-progesterone therapy. 

Within ibandronate therapy, ApaI polymorphism was significantly associated with the FBMD, LBMD, and OC level (Table 7). However, while the subjects with AA and Aa genotypes showed significant BMD improvements, those with aa genotype had no increase in either FBMD or LBMD. The differences in FBMD t-scores comparing AA and Aa genotypes (relative to aa genotype) were 0.442 ± 0.125 (*p* < 0.05) and 0.339 ± 0.116 (*p* < 0.01), respectively. For the LBMD t-score, these comparisons were calculated as 0.496 ± 0.114 (*p* < 0.01) and 0.474 ± 0.106 (*p* < 0.01) for AA and Aa, respectively. All genotypes reduced OC during therapy with effects ranging from 0.788 ± 0.114 µg/L (Aa) to 1.046 ± 0.129 µg/L (aa). The genotypes of TaqI polymorphism were associated with changes in FBMD, LBMD, OC, and β-CTx levels. Significant improvements in FBMD were found for Tt and tt genotypes; LBMD, OC, and β-CTx were improved in all genotypes. In relation to BMD, patients with TT genotype had no response (FBMD) or a weaker but significant response (LBMD) to the therapy. The greatest improvement in these parameters was shown by tt genotype, with an increase in t-score of 0.421 ± 0.101 and 0.914 ± 0.110 for FBMD and LBMD, respectively. Similarly, OC and β-CTx levels were reduced to the highest extent in the tt genotype. Within *VDR*/BsmI polymorphism, improvements in FBMD, LBMD, OC, and β-CTx were found in all genotypes, except for bb genotype in relation to FBMD, where no improvement was determined. Increases in t-score ranged from 0.2 to 0.4 (for FBMD) and up to 0.9 (for LBMD). The differences in FBMD t-score compared to bb genotype were 0.333 ± 0.101 and 0.386 ± 0.128 for BB and Bb genotypes (*p* < 0.01), respectively. For the Cdx polymorphism, genotypes only differed in sP concentration. AA genotypes showed lower sP levels in comparison with GG (0.143 ± 0.048; *p* < 0.05) and GA (0.126 ± 0.047; *p* < 0.05) genotype carriers. 

In raloxifene-treated women, only *VDR*/TaqI and *VDR*/BsmI genotypes were significantly associated with some OP parameters (Table 8). All genotypes of both polymorphisms resulted in improved LBMD, with t-score increases ranging from 0.26 to 0.82. In addition, TaqI polymorphism affected OC level and BsmI polymorphism was associated with β-CTx improvement. The highest reduction of these parameters was achieved by heterozygous genotypes Tt and Bb for OC and β-CTx, respectively. No changes were detected in the other monitored traits in relation to raloxifene therapy. 

Summary graphs presenting the effects estrogen-progesterone, ibandronate, and raloxifene therapies on osteoporosis-related traits across *VDR* genotypes are provided in the supplementary file (see Supplementary file/Figures S1–S3).

## 4. Discussion

Bone as a metabolically active tissue undergoes continuous processes of bone formation and resorption influenced by combined interaction of multiple genes and environmental factors. The identification of gene variants responsible for reduced bone mass has the potential to reveal individuals with increased risk of OP and suggest a personalized clinical approach to prevent the development of this disorder [35].

The first part of our study contributes to the knowledge of the effects of *VDR* gene on BMD, bone turnover markers, biochemical parameters, and fracture prevalence in Slovak postmenopausal women. The frequencies of all genotypes and alleles showed a distribution similar to other Caucasian populations. Several studies confirmed that heterozygous genotypes predominate in the European population [31,35,36,37] and that genotype and allele frequencies may differ from other populations in the world or between ethnic groups due to the evolutionary process and the genetic bases of population behavior [15]. Considering our results, the comparison with studies examining populations from northern Slovakia and Hungary is particularly interesting. Postmenopausal women in our study came from the Slovak southern region which was an important Hungarian-Slavic contact zone for more than a thousand years [38]. However, despite different cultural, linguistic, and geographical origins, allele frequencies for all polymorphisms corresponded in our study with those obtained from northern Slovakia [39] and Hungary [40].

On the other hand, genetic association studies in OP often bring inconsistent findings, especially due to genetic heterogeneity, population admixture, gene-environment, and gene–gene interactions [13]. ApaI polymorphism in the *VDR* gene is located in intron 8 in the 3’-regulatory region, resulting in changes of vitamin D biological activity. In our study, an association between *VDR*/ApaI polymorphism and BMD was not confirmed. We found a significant effect of this polymorphism on OC concentration with higher levels of OC in genotype aa. Consistent with our results, no significant association between *VDR*/ApaI polymorphism and BMD was observed in several studies, including European populations [41,42,43]. In contrast, the association of this polymorphism with BMD was reported in some Asian populations [44,45]. Inconsistent results have been reported in relation to OC. Lorentzon et al. [46] found higher OC levels in subjects with genotype aa, Morrison et al. [47] revealed the opposite effect. Some studies have not confirmed any relationship between *VDR*/ApaI polymorphism and OC level [48,49,50,51]. 

TaqI polymorphism is located in exon 9 of the *VDR* gene. Although this polymorphism produces a silent mutation, it has been shown to influence mRNA stability, leading to protein level changes [52,53]. In our study, women with TT genotype had significantly elevated levels of all bone turnover markers (bALP, OC, β-CTx) compared to genotype Tt, demonstrating an increased rate of remodeling. However, this dynamic was not reflected in a significant reduction of BMD, although both FBMD and LBMD were insignificantly lower versus Tt genotype. Increased β-CTx levels was also found in tt genotype carriers. In Czech postmenopausal women, Zajícková et al. [43] did not reveal the influence of TaqI polymorphism either on BMD or on biochemical markers level. Other studies also found no association with BMD [49,54,55,56]. On the other hand, t allele was associated with low BMD in several studies [41,57,58].

BsmI polymorphism is located in the 3’ untranslated region and is involved in the regulation of mRNA stability. We found an effect of the polymorphism on LBMD, with bb genotype having lower t-score values at this site compared to Bb genotype. The decrease in LBMD was accompanied by an increase in bone turnover markers (OC and β-CTx) and a reduction of sP. These findings point to the riskiness of bb genotype in relation to elevated bone remodeling rate, leading to decreased LBMD. The results of other studies suggest a rather negative effect of B allele on FBMD [41,57] or even on LBMD in 20–40-year-old women [59]. On the other hand, some studies as well as meta-analyses did not confirm the impact of BsmI polymorphism on BMD [43,54,55,56,60,61,62,63]. However, in a recent meta-analysis, this polymorphism was connected with the risk of OP in Caucasians [64]. It is possible that Ca intake may affect the effect of BsmI polymorphism on BMD. In the study by Macdonald et al. [65], homozygotes for b allele had greater BMD compared to carriers of B allele at low Ca intake. No difference was observed with increasing Ca intake.

*VDR*/Cdx2 variant is located in the 5′-promoter region of the *VDR* gene, with G allele reducing the transcriptional activity of the gene to 70% of A allele [66]. We found the significant effect of *VDR*/Cdx2 polymorphism only on bALP concentration. The Cdx2 polymorphism was associated with BMD in Japanese population [66,67] and a relationship between Cdx2 and FBMD was observed in osteoporotic postmenopausal and premenopausal women in Caucasian population [19]. Cdx2 was also associated with total hip BMD in postmenopausal women from eastern Slovakia [68].

FokI polymorphism (C/T transition) is located in the start codon of the *VDR* gene. It is known to affect VDR polypeptide chain structure by an alternative translation start site leading to two isoforms of *VDR* [69,70]. Our findings did not show significant associations of FokI polymorphism with osteoporosis-related traits, similarly to postmenopausal women from eastern Slovakia [68]. On the other hand, Zajícková et al. [43] found significant differences between the genotypes with the ff genotype having a lower BMD in Czech postmenopausal women. In general, FokI polymorphism was associated with BMD in postmenopausal Asian women [64,71]. A recent meta-analysis revealed a significant association in the dominant model of FokI (ff + Ff vs. FF) in an overall analysis, including Asian, Caucasian, and other populations; however, no significant association was found in the Caucasian subgroup [72].

It should also be noted that although the findings of some studies do not provide evidence for the effect of individual polymorphisms on BMD, several of them point to their possible effect due to interactions. For example, the haplotypes AaBb [37] and BBAAtt [17] were associated with an increased risk of OP, while the haplotype BbaaTT was described as a protective factor against OP in Spanish population [17]. In postmenopausal Indian women, the presence of T allele (TaqI) in combination with aa (ApaI) and bb (BsmI) showed positive influence on BMD [44]. However, analysis of haplotypes and genotype combinations in our study could not be performed due to the low number of samples.

Our study found associations of fracture incidence with ApaI, TaqI, and BsmI genotypes. Carriers of Apal/aa, TaqI/TT, and BsmI/bb genotypes had a two to three times higher chance of suffering spinal, radial, or total fractures. In BsmI polymorphism, the findings were supported by a lower LBMD in bb genotype. In previous years, several studies and meta-analyses reported the association of *VDR* polymorphisms with osteoporotic fractures [62,73,74,75]. However, results were inconsistent, with differences in sample size, ethnicity, and sampling methods used. Some of them [73] revealed the same risk alleles for the occurrence of fractures as in our study, while others described the association of opposite alleles with fractures [62] and the remaining studies did not reveal any relationship between *VDR* polymorphisms and the presence of fractures [75]. In the meta-analysis by Mu et al. [76], ApaI polymorphism did not significantly affect the risk of osteoporotic fracture; however, after racial stratification, genotype aa elevated the risk of osteoporotic fractures in European countries. No significant associations between the remaining *VDR* polymorphisms (BsmI, TaqI, FokI, and Cdx2) and osteoporotic fracture were found. In several studies, the relationship between fracture incidence were independent of BMD at the respective fracture site. This suggests, that genetic variability may contribute to fracture risk also through a mechanism other than bone mass. Determinants, such as bone size and shape, geometry, cortical porosity and thickness, trabecular bone morphology, bone micro-damages, and quality of bone tissue, can also contribute to fragility fractures [71].

The last part of our study examined the response to three types of antiresorptive therapy in relation to *VDR* genotypes. The effect of all analyzed treatment types positively influenced FBMD, LBMD and also biochemical markers. Considering the *VDR* gene, data evaluation revealed the impact of *VDR* polymorphisms on ibandronate and raloxifene treatment efficiency. In general, several studies emphasize the *VDR* gene as an important factor influencing the effectiveness of antiosteoporotic treatment. *VDR* gene polymorphisms may modify the BMD response to Ca intake, Ca and vitamin D supplementation, or hormone replacement therapy [77]. The action of ibandronate on bone tissue is based on its inhibition of osteoclast activity, bone resorption, and turnover [24]. SERMs have the ability to bind to the estrogen receptor and act as a receptor agonist or antagonist in a tissue-specific manner [78]. Raloxifene is an estrogen receptor agonist in bone and it was the first SERM approved to prevent bone loss in women with postmenopausal OP [79]. There is also a link between 17β-estradiol and VDR. Estrogen is able to regulate the transcription and expression of the *VDR* gene, and to increase the number of VDR in the osteoblast-like cell line associated with enhanced responsiveness of the cells to vitamin D [80]. Based on our findings with ibandronate therapy, ApaI/aa, TaqI/TT, and BsmI/bb genotypes may be considered to be less responsive to the therapy. In addition, changes in OC and β-CTx levels were determined, where carriers with aa, TT, and bb genotypes had a smaller decrease in marker levels compared to others genotypes. Under raloxifene therapy, LBMD increased significantly more in women homozygous for tt (TaqI) and BB (BsmI) genotypes. Our results indicate that genetic polymorphisms in the *VDR* gene may influence the efficacy of ibandronate and raloxifene treatment and should be considered when recommending that treatment. Associations between antiosteoporotic treatment and *VDR* polymorphic variants have been investigated in several studies. Among the studied polymorphisms, BsmI genotypes were demonstrated to influence the efficacy of alendronate and hormone replacement therapy in postmenopausal osteoporotic women [81]. Similar results were obtained by Creatsa et al. [82] using alendronate treatment. However, in both studies, carriers of b allele responded better to the treatment. On the other hand, BsmI polymorphism was not associated with alendronate therapy in postmenopausal women from southern Italy [83]. Conversely, an association with FokI polymorphism was found in this study, with ff genotype carriers being the best responders. The exact mechanism by which the *VDR* gene polymorphisms affects treatment efficiency remains unknown. Perhaps transcriptional and/or post-transcriptional regulation by VDR activation could cause alteration in genes involved in the drug metabolism, which may lead to pharmacokinetic or pharmacodynamics consequences for different drug response in clinical use [84].

## 5. Conclusions

This study provides further support for a significant role of *VDR* gene polymorphisms in bone metabolism and risk of postmenopausal OP and osteoporotic fractures. It also points to the important function of *VDR* genotypes in response to antiresorptive treatment with ibandronate and raloxifene, and provides a basis for predicting the efficiency of these therapies. Of the osteoporosis-related traits, only LBMD was influenced by BsmI polymorphism. Four polymorphisms (ApaI, BsmI, TaqI, and Cdx2) were associated with bone turnover markers. ApaI, TaqI, and BsmI genotypes also increased the risk of spinal, radial, or total fractures. Pharmacogenetic evaluation revealed three genotypes (ApaI/aa, TaqI/TT, and BsmI/bb) showing a weaker or no response to ibandronate therapy. Two polymorphisms (TaqI and BsmI) were associated with raloxifene therapy. More attention should be devoted to investigating the underlying mechanisms and causal causes of the positive associations observed between *VDR* polymorphisms and osteoporosis-related traits, as well as the efficacy of antiresorptive treatment.

## Figures and Tables

**Table 1:** Polymerase chain reaction restriction fragment length polymorphism (PCR-RFLP) conditions for detection of vitamin D receptor single nucleotide polymorphisms.

Polymorphism	PCR Product Size (bp)	Restriction Endonuclease	Base Change	Restriction Site vs. Genotype Nomenclature
Apa (rs7975232)	740	Bsp120I	G_wt_/T_mut_	TT GT GG vs. AA Aa aa
TaqI (rs731236)	740	TaqI	T_wt_/C_mut_	TT TC CC vs. TT Tt tt
BsmI (rs1544410)	825	Mva 1269I	G_wt_/A_mut_	AA GA GG vs. BB Bb bb
FokI (rs2228570)	265	FokI	C_wt_/T_mut_	CC CT TT vs. FF Ff ff
Cdx2 (rs11568820)	250	Bpu10I	G_wt_/A_mut_	AA GA GG vs. CC Cc cc

wt—wild type allele, mut—mutated allele.

**Table 2:** Distribution of *VDR* genotypes and alleles.

Polymorphism	Genotype	Number	Genotype Frequency (%)	HWE *p* Value	Allele Frequency
rs7975232 (ApaI)	AA	80	24	χ^2^ = 0.59 *p* = 0.75	A = 0.5
	Aa	176	52		a = 0.5
	aa	82	24		
rs731236 (TaqI)	TT	123	39	χ^2^ = 0.058 *p* = 0.97	T = 0.63
	Tt	150	47		t = 0.37
	tt	43	14		
rs1544410 (BsmI)	BB	48	15	χ^2^ = 0.014	B = 0.38
	Bb	154	47	*p* = 0.99	b = 0.62
	bb	127	38		
rs228570 (FokI)	FF	92	31	χ^2^ = 5.36 *p* = 0.07	F = 0.6
	Ff	160	55		f = 0.4
	ff	40	13		
rs11568820 (Cdx2)	GG	155	45	χ^2^ = 0.112	G = 0.67
	GA	148	43	*p* = 0.95	A = 0.33
	AA	39	11		

HWE—Hardy–Weinberg equilibrium (the chi-square test value).

**Table 3:** General characteristics of the studied groups of women.

Variable	Total N = 356	EPT Study N = 76	Raloxifene Study N = 64	Ibandronate Study N = 74
Age (years)	63.13 ± 0.45	63.22 ± 1.00	65.30 ± 0.98	63.30 ± 0.87
Body mass index (BMI)	27.51 ± 0.07	27.30 ± 0.18	27.64 ± 0.17	28.16 ± 0.16
FBMD (t-score)	−1.82 ± 0.03	−2.13 ± 0.04	−2.16 ± 0.06	−2.03 ± 0.06
LBMD (t-score)	−2.44 ± 0.04	−2.87 ± 0.04	−2.95 ± 0.05	−2.91 ± 0.04
bALP (μkat/L)	0.56 ± 0.03	1.17 ± 0.11	0.68 ± 0.08	0.42 ± 0.05
OC (μg/L)	3.93 ± 0.05	3.92 ± 0.11	4.25 ± 0.13	4.24 ± 0.11
β-CTx (ng/L)	722.07 ± 12.54	795.23 ± 24.21	876.86 ± 28.06	853.62 ± 24.89
sCa (mmol/L)	2.40 ± 0.01	2.39 ± 0.02	2.46 ± 0.03	2.45 ± 0.02
sP (mmol/L)	1.12 ± 0.01	1.19 ± 0.02	1.23 ± 0.03	1.21 ± 0.02

Data are presented as Mean ± SE (SE—standard error of the mean); FBMD—femoral neck bone mineral density; LBMD—lumbar spine bone mineral density; bALP—bone isoenzyme of alkaline phosphatase; OC—osteocalcin; β-CTx—BetaCrosslaps; sCa—serum calcium; sP—serum phosphate; EPT—hormone therapy (17β-estradiol/progesterone).

**Table 4:** Associations of the *VDR* gene polymorphisms with osteoporosis in postmenopausal women.

Parameter	ApaI/AA N = 80	ApaI/Aa N = 176	ApaI/aa N = 82	Sig. *p* Value
FBMD	−1.736 ± 0.057	−1.753 ± 0.038	−1.854 ± 0.056	NS
LBMD	−2.337 ± 0.080	−2.309 ± 0.054	−2.2438 ± 0.079	NS
bALP	0.540 ± 0.073	0.498 ± 0.050	0.631 ± 0.072	NS
OC	3.818 ± 0.110	3.677 ± 0.074	4.101 ± 0.108	Aa−aa: *p* = 0.001
β-CTx	696.279 ± 27.364	689.964 ± 18.475	745.411 ± 26.981	NS
sCa	2.372 ± 0.022	2.402 ± 0.015	2.403 ± 0.022	NS
sP	1.197 ± 0.020	1.204 ± 0.013	1.182 ± 0.019	NS
**Parameter**	**Taq/TT** **N = 123**	**Taq/Tt** **N = 150**	**Taq/tt** **N = 43**	**Sig.** ***p* value**
FBMD	−1.821 ± 0.046	−1.731 ± 0.042	−1.795 ± 0.079	NS
LBMD	−2.421 ± 0.063	−2.257 ± 0.057	−2.444 ± 0.107	NS; *p* = 0.055
bALP	0.619 ± 0.057	0.451 ± 0.052	0.538 ± 0.097	TT−Tt: *p* = 0.030
OC	4.007 ± 0.089	3.743 ± 0.080	3.747 ± 0.150	TT−Tt: *p* = 0.029
β-CTx	736.481 ± 21.852	671.661 ± 19.778	757.663 ± 36.964	TT−Tt: *p* = 0.029 Tt−tt: *p* = 0.041
sCa	2.403 ± 0.018	2.402 ± 0.016	2.360 ± 0.030	NS
sP	1.196 ± 0.024	1.207 ± 0.014	1.188 ± 0.015	NS
**Parameter**	**BsmI/BB** **N = 48**	**BsmI/Bb** **N = 154**	**BsmI/bb** **N = 127**	**Sig.** ***p* value**
FBMD	−1.793 ± 0.074	−1.751 ± 0.041	−1.838 ± 0.046	NS
LBMD	−2.465 ± 0.101	−2.276 ± 0.056	−2.443 ± 0.062	Bb−bb: *p* = 0.047
bALP	0.526 ± 0.096	0.507 ± 0.054	0.613 ± 0.059	NS
OC	3.809 ± 0.144	3.767 ± 0.081	4.031 ± 0.089	Bb−bb: *P*=0.028
β-CTx	754.169 ± 35.360	677.053 ± 19.745	739.780 ± 21.751	Bb−bb: *p* = 0.034
sCa	2.375 ± 0.029	2.402 ± 0.016	2.411 ± 0.018	NS
sP	1.253 ± 0.025	1.197 ± 0.014	1.184 ± 0.015	BB−Bb: *p* = 0.048 BB−bb: *p* = 0.018
**Parameter**	**Cdx2/GG** **N = 155**	**Cdx2/GA** **N = 148**	**Cdx2/AA** **N = 39**	**Sig.** ***p* value**
FBMD	−1.810 ± 0.041	−1.817 ± 0.042	−1.713 ± 0.082	NS
LBMD	−2.398 ± 0.057	−2.413 ± 0.058	−2.280 ± 0.113	NS
bALP	0.646 ± 0.053	0.512 ± 0.054	0.385 ± 0.106	GG−AA: *p* = 0.027
OC	3.933 ± 0.081	3.743 ± 0.083	3.892 ± 0.161	NS
β-CTx	718.361 ± 19.720	723.313 ± 20.220	694.948 ± 39.408	NS
sCa	2.387 ± 0.016	2.409 ± 0.016	2.416 ± 0.032	NS
sP	1.201 ± 0.014	1.206 ± 0.014	1.186 ± 0.028	NS
**Parameter**	**FokI/FF** **N = 92**	**FokI/Ff** **N = 160**	**FokI/ff** **N = 40**	**Sig.** ***p* value**
FBMD	−1.752 ± 0.055	−1.741 ± 0.041	−1.684 ± 0.087	NS
LBMD	−2.310 ± 0.058	−2.272 ± 0.058	−2.379 ± 0.116	NS
bALP	0.460 ± 0.069	0.606 ± 0.052	0.440 ± 0.105	NS
OC	3.698 ± 0.104	3.805 ± 0.079	3.942 ± 0.160	NS
β-CTx	676.145 ± 25.336	691.105 ± 19.242	701.075 ± 38.811	NS
sCa	2.386 ± 0.021	2.383 ± 0.016	2.433 ± 0.032	NS
sP	1.213 ± 0.018	1.183 ± 0.014	1.203 ± 0.028	NS

Data are presented as Estimated Marginal Mean ± SE (SE—standard error of the mean), values are adjusted for age and BMI; FBMD—femoral neck BMD (t-score); LBMD—lumbar spine BMD (t-score); bALP—bone isoenzyme of alkaline phosphatase (μkat/L); OC—osteocalcin (μg/L); β-CTx—BetaCrosslaps (ng/L); sCa—serum calcium (mmol/L); sP—serum phosphate (mmol/L); Sig.—significance, NS—non-significant differences; *p* values determine significant differences (*p* < 0.05).

**Table 5:** The effects of the *VDR* genotypes on fracture prevalence.

Fracture Location	SNP	Genotype	Presence of Fractures (N)	Absence of Fractures (N)	*p* Value	OR	95% CI
Spinal fractures	ApaI	AA	16	64	AA-aa: 0.089	2.019	0.898–4.542
		Aa	40	136	Aa-aa: 0.027 *	2.150	1.090–4.242
		aa	28	54	Aa-AA: 0.870	1.065 ^b^	0.504–2.249
	TaqI	TT	39	84	TT-tt: 0.771	0.870	0.341–2.221
		Tt	28	122	Tt-tt: 0.134	2.076	0.800–5.390
		tt	11	32	Tt-TT: 0.01 **	2.386 ^b^	1.230–4.630
	BsmI	BB	12	36	BB-bb: 0.350	1.514	0.634–3.617
		Bb	30	124	Bb-bb: 0.005 **	2.481	1.316–4.677
		bb	44	83	Bb-BB: 0.273	1.638 ^b^	0.677–3.963
	Cdx2	GG	40	115	GG-AA: 0.710	1.194	0.470–3.031
		GA	41	107	GA-AA: 0.961	1.023	0.403–2.595
		AA	10	29	GA-GG: 0.610	0.857 ^b^	0.474–1.550
	FokI	FF	20	72	FF-ff: 0.419	1.483	0.571–3.851
		Ff	29	131	Ff-ff: 0.151	1.913	0.789–4.635
		ff	13	27	Ff-FF: 0.498	1.290 ^b^	0.617–2.697
Radial fractures	ApaI	AA	12	68	AA-aa: 0.167	1.822	0.778–4.270
		Aa	19	157	Aa-aa: 0.003 **	3.173	1.487–6.773
		aa	21	61	Aa-AA: 0.198	1.741 ^b^	0.748–4.053
	TaqI	TT	23	100	TT-tt: 0.730	0.832	0.293–2.360
		Tt	19	131	Tt-tt: 0.628	1.297	0.453–3.712
		tt	16	37	Tt-TT: 0.219	1.558 ^b^	0.768–3.160
	BsmI	BB	7	41	BB-bb: 0.493	1.403	0.532–3.700
		Bb	22	132	Bb-bb: 0.207	1.537	0.788–2.996
		bb	27	100	Bb-BB: 0.856	1.095 ^b^	0.412–2.908
	Cdx2	GG	26	129	GG-AA: 0.838	0.892	0.299–2.662
		GA	26	122	GA-AA: 0.739	0.830	0.278–2.483
		AA	5	34	GA-GG: 0.827	0.931 ^b^	0.488–1.773
	FokI	FF	13	79	FF-ff:0.732	1.209	0.408–3.586
		Ff	20	140	Ff-ff: 0.622	1.288	0.471–3.526
		ff	7	33	Ff-FF: 0.879	1.065 ^b^	0.471–2.408
Total fractures	ApaI	AA	22	58	AA-aa: 0.237	1.564	0.744–3.289
		Aa	42	134	Aa-aa: 0.007 **	2.434	1.273–4.654
		aa	32	50	Aa-AA: 0.206	1.556 ^b^	0.785–3.086
	TaqI	TT	23	100	TT-Tt: 0.983	1.010	0.424–2.404
		Tt	19	131	Tt-tt: 0.139	1.933	0.807–4.628
		tt	6	37	Tt-TT: 0.037 *	1.915 ^b^	1.040–3.526
	BsmI	BB	15	48	BB-bb: 0.721	1.159	0.517–2.595
		Bb	36	154	Bb-bb: 0.018 **	2.034	1.128–3.668
		bb	47	127	Bb-BB: 0.173	1.756 ^b^	0.781–3.946
	Cdx2	GG	46	109	GG-AA: 0.848	1.090	0.452–2.628
		GA	46	102	GA-AA: 0.985	0.992	0.411–2.394
		AA	11	28	GA-GG: 0.959	0.982 ^b^	0.522–1.587
	FokI	FF	21	71	FF-ff: 0.304	1.610	0.650–3.992
		Ff	36	124	Ff-ff: 0.279	1.582	0.690–3.628
		ff	14	26	Ff-FF: 0.739	0.910 ^b^	0.495–1.950

Total fractures count a presence of any fracture of an individual; SNP—single nucleotide polymorphism; *p* values determine significant differences (* *p* < 0.05; ** *p* < 0.01); OR—odds ratio, b—genotypes AA, TT, BB, GG, and FF were set as baseline categories in the regression model; otherwise, the baseline categories were represented by genotypes aa, tt, bb, AA, and ff; CI—confidence interval; femoral fractures were not evaluated.

**Table 6:** The effect of treatment types on osteoporosis-related traits.

Treatment Type	Osteoporosis Related Traits	Value before Treatment	Value after Treatment	Differences after 48 Months	Sig. *p* Value
EPT	FBMD	−2.132 ± 0.044	−1.7842 ± 0.040	0.34737 ± 0.043	<0.001
	LBMD	−2.871 ± 0.044	−2.1842 ± 0.048	0.68684 ± 0.057	<0.001
	bALP	1.1645 ± 0.109	0.8753 ± 0.083	0.28921 ± 0.049	<0.001
	OC	3.9179 ± 0.114	3.1220 ± 0.089	0.79592 ± 0.090	<0.001
	β-CTx	795.2276 ± 24.209	519.1579 ± 21.812	276.06974 ± 22.583	<0.001
	sCa	2.3904 ± 0.023	2.3142 ± 0.016	0.07618 ± 0.023	0.006
	sP	1.1912 ± 0.017	1.1167 ± 0.017	0.07447 ± 0.021	0.001
Ibandronate	FBMD	−2.027 ± 0.057	−1.867 ± 0.049	0.160 ± 0.047	0.001
	LBMD	−2.915 ± 0.043	−2.405 ± 0.060	0.509 ± 0.054	<0.001
	bALP	0.424 ± 0.048	0.330 ± 0.032	0.094 ± 0.024	<0.001
	OC	4.241 ± 0.109	3.309 ± 0.083	0.933 ± 0.069	<0.001
	β-CTx	853.623 ± 24.894	555.931 ± 21.763	297.692 ± 20.283	<0.001
	sCa	2.450 ± 0.023	2.389 ± 0.023	0.061 ± 0.033	NS
	sP	1.213 ± 0.019	1.194 ± 0.020	0.020 ± 0.026	NS
Raloxifene	FBMD	−2.155 ± 0.059	−1.913 ± 0.059	−0.242 ± 0.070	0.001
	LBMD	−2.947 ± 0.054	−2.484 ± 0.070	0.462 ± 0.063	<0.001
	bALP	0.679 ± 0.081	0.561 ± 0.068	0.119 ± 0.034	0.001
	OC	4.251 ± 0.130	3.466 ± 0.107	0.785 ± 0.090	<0.001
	β-CTx	876.860 ± 28.059	620.830 ± 24.873	256.030 ± 20.840	<0.001
	sCa	2.463 ± 0.025	2.347 ± 0.021	0.115 ± 0.033	0.001
	sP	1.255 ± 0.023	1.134 ± 0.020	0.091 ± 0.032	0.006

Data are presented as Estimated Marginal Mean ± SE (SE—standard error of the mean); FBMD—femoral neck BMD (t-score); LBMD—lumbar spine BMD (t-score); bALP—bone isoenzyme of alkaline phosphatase (μkat/L); OC—osteocalcin (μg/l); β-CTx—BetaCrosslaps (ng/L); sCa—serum calcium (mmol/L); sP—serum phosphate (mmol/L); EPT—combined estrogen-progesterone hormone therapy (17β-estradiol/progesterone); Sig.—significance of variable difference after treatment, *p* values determine significant differences (*p* < 0.05), NS—non-significant difference.

**Table 7:** Significant changes in osteoporosis-related traits after ibandronate therapy in relation to the *VDR* genotypes.

SNP	Analyzed Trait	Genotypes	Before Treatment	After Treatment	Mean Difference within or between Genotypes	Sig. (*p* Value); Treatment Effect	Sig. (*p* Value); Genotype Effect
ApaI	FBMD (t-score)	AA	−1.925 ± 0.108	−1.605 ± 0.089	0.320 ± 0.086	<0.001	
		Aa	−1.989 ± 0.093	−1.748 ± 0.077	0.241 ± 0.074	0.002	
		aa	−2.167 ± 0.105	−2.248 ± 0.087	0.081 ± 0.084	NS	
		AA-Aa			0.104 ± 0.118		NS
		AA-aa			0.442 ± 0.125		0.002
		Aa-aa			0.339 ± 0.116		0.013
	LBMD	AA	−2.870 ± 0.079	−2.108 ± 0.109	0.765 ± 0.098	0.002	
	(t-score)	Aa	−2.816 ± 0.069	−2.213 ± 0.092	0.765 ± 0.098	0.002	
		aa	−3.054 ± 0.080	−2.842 ± 0.107	0.167 ± 0.096	0.002	
		AA-Aa			0.022 ± 0.107		NS
		AA-aa			0.496 ± 0.114		<0.001
		Aa-aa			0.474 ± 0.106		<0.001
	OC	AA	4.099 ± 0.192	3.064 ± 0.145	−1.035 ± 0.133	<0.001	
		Aa	3.929 ± 0.166	3.141 ± 0.124	−0.788 ± 0.114	<0.001	
		aa	4.775 ± 0.188	3.730 ± 0.141	−1.046 ± 0.129	<0.001	
		AA-Aa			0.046 ± 0.206		NS
		AA-aa			−0.671 ± 0.219		0.009
		Aa-aa			−0.717 ± 0.203		0.002
TaqI	FBMD	TT	−2.144 ± 0.099	−2.174 ± 0.077	−0.030 ± 0.073	NS	
	(t-score)	Tt	−1.981 ± 0.099	−1.707 ± 0.077	0.274 ± 0.073	<0.001	
		tt	−1.921 ± 0.014	−1.500 ± 0.107	0.421 ± 0.101	<0.001	
		TT-Tt			−0.315 ± 0.114		0.020
		TT-tt			−0.449 ± 0.138		0.005
		Tt-tt			−0.134 ± 0.138		NS
	LBMD (t-score)	TT	−3.011 ± 0.073	−2.822 ± 0.096	0.189 ± 0.079	0.001	
		Tt	−2.870 ± 0.073	−2.237 ± 0.096	0.633 ± 0.079	0.001	
		tt	−2.836 ± 0.101	−1.921 ± 0.133	0.914 ± 0.110	0.001	
		TT-Tt			−0.363 ± 0.106		0.003
		TT-tt			−0.538 ± 0.128		0.000
		Tt-tt			−0.175 ± 0.128		NS
	OC	TT	4.203 ± 0.215	2.959 ± 0.154	−0.939 ± 0.116	<0.001	
		Tt	3.792 ± 0.165	3.072 ± 0.119	−0.774 ± 0.116	<0.001	
		tt	4.721 ± 0.160	3.764 ± 0.115	−1.190 ± 0.161	<0.001	
		TT-Tt			0.736 ± 0.188		0.001
		TT-tt			0.703 ± 0.227		0.008
		Tt-tt			0.033 ± 0.227		NS
	β-CTx	TT	893.900 ± 39.930	656.256 ± 31.487	−237.044 ± 32.447	<0.001	
		Tt	790.019 ± 39.930	486.581 ± 31.487	−303.437 ± 32.447	<0.001	
		tt	903.014 ± 55.452	484.221 ± 43.727	−418.793 ± 45.061	<0.001	
		TT-Tt			136.478 ± 45.380		0.010
		TT-tt			81.160 ± 54.913		NS
		Tt-tt			−55.318 ± 54.913		NS
BsmI	FBMD	BB	−1.981 ± 0.125	−1.556 ± 0.099	0.425 ± 0.093	<0.001	
	(t-score)	Bb	−1.941 ± 0.096	−1.704 ± 0.076	0.237 ± 0.072	<0.001	
		bb	−2.138 ± 0.093	−2.172 ± 0.074	0.034 ± 0.069	NS	
		BB-Bb			0.054 ± 0.129		NS
		BB-bb			0.386 ± 0.128		0.010
		Bb-bb			0.333 ± 0.101		0.009
	LBMD	BB	−2.894 ± 0.092	−1.981 ± 0.123	0.913 ± 0.102	<0.001	
	(t-score)	Bb	−2.826 ± 0.071	−2.237 ± 0.095	0.589 ± 0.078	<0.001	
		bb	−3.010 ± 0.068	−2.807 ± 0.091	0.203 ± 0.075	0.009	
		BB-Bb			0.094 ± 0.121		NS
		BB-bb			0.471 ± 0.119		0.001
		Bb-bb			0.377 ± 0.102		0.001
	OC	BB	4.203 ± 0.215	2.959 ± 0.154	−1.244 ± 0.144	<0.001	
		Bb	3.792 ± 0.165	3.072 ± 0.119	−0.720 ± 0.111	<0.001	
		bb	4.721 ± 0.160	3.764 ± 0.115	−0.956 ± 0.107	<0.001	
		BB-Bb			0.149 ± 0.218		NS
		BB-bb			−0.662 ± 0.215		0.008
		Bb-bb			−0.811 ± 0.185		<0.001
	β-CTx	BB	928.313 ± 51.221	491.287 ± 41.383	−437.025 ± 40.255	<0.001	
		Bb	766.619 ± 39.430	481.096 ± 31.857	−285.522 ± 30.988	<0.001	
		bb	904.583 ± 38.046	670.228 ± 30.739	−234.355 ± 29.901	<0.001	
		BB-Bb			85.943 ± 52.987		NS
		BB-bb			−77.605 ± 52.303		NS
		Bb-bb			−163.548 ± 44.915		0.001
Cdx2	sP	GG	1.241 ± 0.029	1.213 ± 0.033	−0.028 ± 0.044	NS	
		GA	1.217 ± 0.027	1.204 ± 0.030	−0.013 ± 0.040	NS	
		AA	1.089 ± 0.0560	1.080 ± 0.063	−0.009 ± 0.083	NS	
		GG-GA GG-AA			0.0167 ± 0.030 0.143 ± 0.048		NS 0.011
		GA-AA			0.126 ± 0.047		0.025

Data are presented expressed as Estimated Marginal Mean ± SE (SE—standard error of the mean); BMD of femoral neck (FBMD) and lumbar spine (LBMD), OC—osteocalcin (μg/L), β-CTx—BetaCrosslaps (ng/L); sP—serum phosphate (mmol/L), Sig.—significance, NS—non-significant differences, *p* values determine significant differences (*p* < 0.05). Only parameters in which differences between *VDR* genotypes were identified are presented.

**Table 8:** Significant changes in osteoporosis-related traits after raloxifene therapy in relation to the *VDR* genotypes.

SNP	Analyzed Trait	Genotypes	Before Treatment	After Treatment	Mean Difference within or between Genotypes	Sig. (*p* Value); Treatment Effect	Sig. (*p* Value); Genotype Effect
TaqI	LBMD (t-score)	TT	−2.936 ± 0.079	−2.6668 ± 0.100	0.268 ± 0.094	0.006	
		Tt	−2.952 ± 0.081	−2.386 ± 0.102	0.567 ± 0.096	<0.001	
		tt	−2.825 ± 0.131	−2.000 ± 0.166	0.825 ± 0.156	<0.001	
		TT-Tt			−0.132 ± 0.110		NS
		TT-tt			0.390 ± 0.149		0.031
		Tt-tt			−0.257 ± 0.150		NS
	OC	TT	4.568 ± 0.219	3.944 ± 0.164	−0.624 ± 0.153	<0.001	
		Tt	4.308 ± 0.232	3.252 ± 0.168	−1.056 ± 0.157	<0.001	
		tt	3.700 ± 0.363	3.014 ± 0.273	−0.686 ± 0.254	0.010	
		TT-Tt			0.476 ± 0.255		NS
		TT-tt			0.899 ± 0.344		0.032
		Tt-tt			0.408 ± 0.360		NS
BsmI	LBMD	BB	−2.844 ± 0.119	−1.989 ± 0.168	0.822 ± 0.157	<0.001	
	(t-score)	Bb	−2.960 ± 0.080	−2.430 ± 0.112	0.530 ± 0.105	<0.001	
		bb	−2.973 ± 0.070	−2.712 ± 0.099	0.262 ± 0.092	0.007	
		BB-Bb			0.295 ± 0.147		NS
		BB-bb			0.442 ± 0.141		0.008
		Bb-bb			0.147 ± 0.109		NS
	β-CTx	BB	764.089 ± 72.016	452.011 ± 64.231	−312.078 ± 50.354	<0.001	
		Bb	954.425 ± 48.310	637.100 ± 43.081	−317.325 ± 33.779	<0.001	
		bb	870.581 ± 42.371	679.328 ± 37.790	−191.253 ± 29.626	<0.001	
		BB-Bb			−187.713 ± 76.368		0.045
		BB-bb			−166.904 ± 73.582		NS
		Bb-bb			20.808 ± 56.588		NS

Data are presented expressed as Estimated Marginal Mean ± SE (SE—standard error of the mean); LBMD—BMD of lumbar spine; OC—osteocalcin (μg/L), β-CTx—BetaCrosslaps (ng/L), Sig.—significance, NS—non-significant differences, *p* values determine significant differences (*p* < 0.05). Only parameters in which differences between *VDR* genotypes were identified are presented.

## Data Availability

The data presented in this study are available on request from the corresponding author.

## Supplementary Materials

The following supporting information can be downloaded at: https://www.mdpi.com/article/10.3390/genes14010193/s1, Figure S1: Effects of EPT therapy on osteoporosis-related traits across *VDR* genotypes. FBMD—femoral neck BMD, LBMD—lumbar spine BMD, bALP—bone isoenzyme of alkaline phosphatase, OC—osteocalcin, β-CTx—BetaCrosslaps, sCa—serum calcium, sP—serum phosphate, P values determine significant difference (*** *p* < 0.001; ** *p* < 0.01, * *p* < 0.05). Figure S2: Effects of ibandronate therapy on osteoporosis-related traits across *VDR* genotypes. FBMD—femoral neck BMD, LBMD—lumbar spine BMD, bALP—bone isoenzyme of alkaline phosphatase, OC—osteocalcin, β-CTx—BetaCrosslaps, sCa—serum calcium, sP—serum phosphate, P values determine significant differences (*** *p* < 0.001; ** *p* < 0.01, * *p* < 0.05). Figure S3: Effects of raloxifene therapy on osteoporosis-related traits across *VDR* genotypes. FBMD—femoral neck BMD, LBMD—lumbar spine BMD, bALP—bone isoenzyme of alkaline phosphatase, OC—osteocalcin, β-CTx—BetaCrosslaps, sCa—serum calcium, sP—serum phosphate, P values determine significant differences (*** *p* < 0.001; ** *p* < 0.01, * *p* < 0.05).
