# Supplementary material for: Vitamin D Receptor Gene Polymorphisms Affect Osteoporosis-Related Traits and Response to Antiresorptive Therapy
Source: Genes (Basel). 2023 Jan 11;14(1):193. doi: 10.3390/genes14010193 (PMC9858724; doi:10.3390/genes14010193)
Supplement: Supplementary file 1 [file genes-14-00193-s001.zip › genes-2132718-supplementary.pdf]

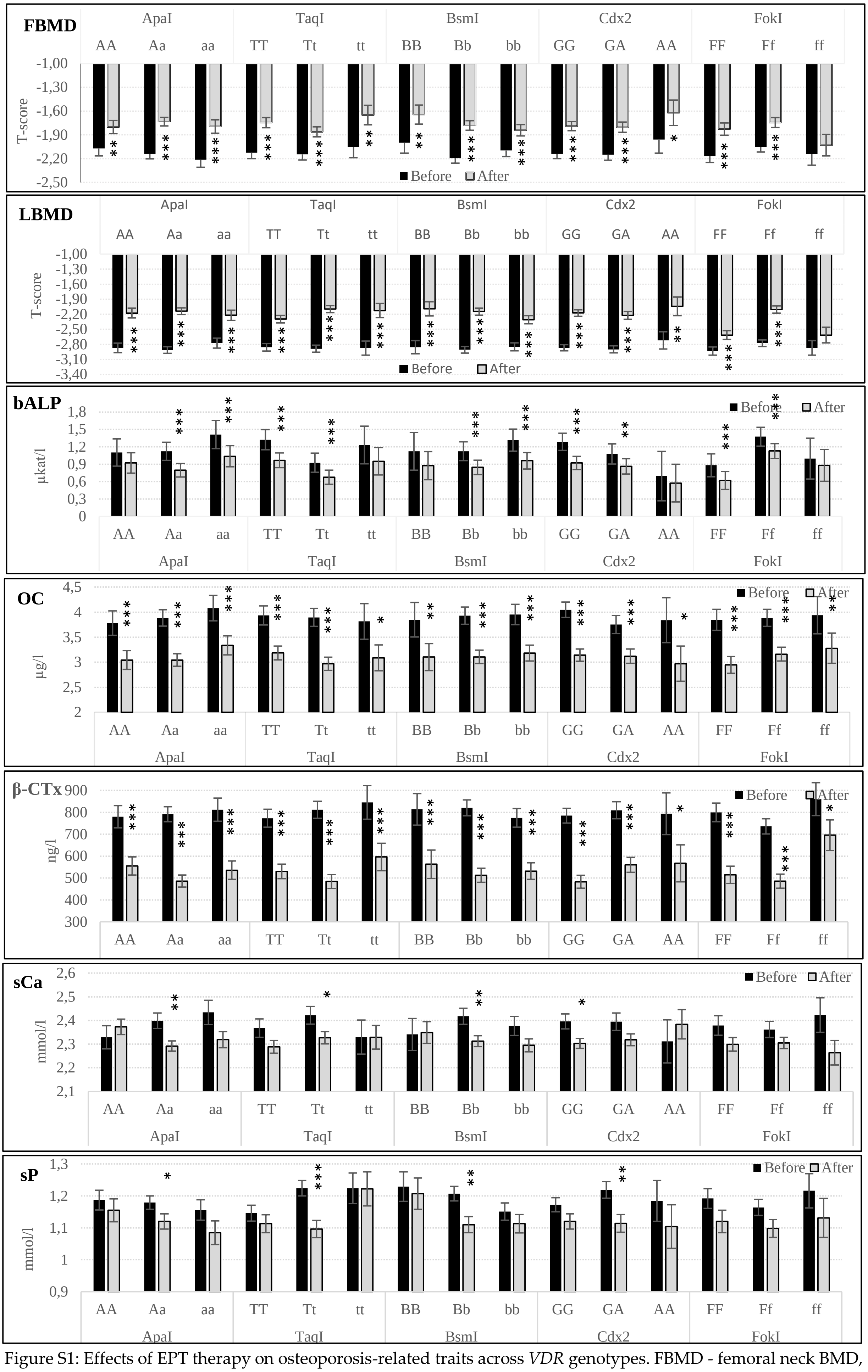

Figure S1: Effects of EPT therapy on osteoporosis-related traits across *VDR* genotypes. FBMD - femoral neck BMD, LBMD - lumbar spine BMD, bALP – bone isoenzyme of alkaline phosphatase, OC – osteocalcin, β-CTx - BetaCrosslaps, sCa - serum calcium, sP - serum phosphate, P values determine significant difference (***P<0.001; **P<0.01, *P<0.05).

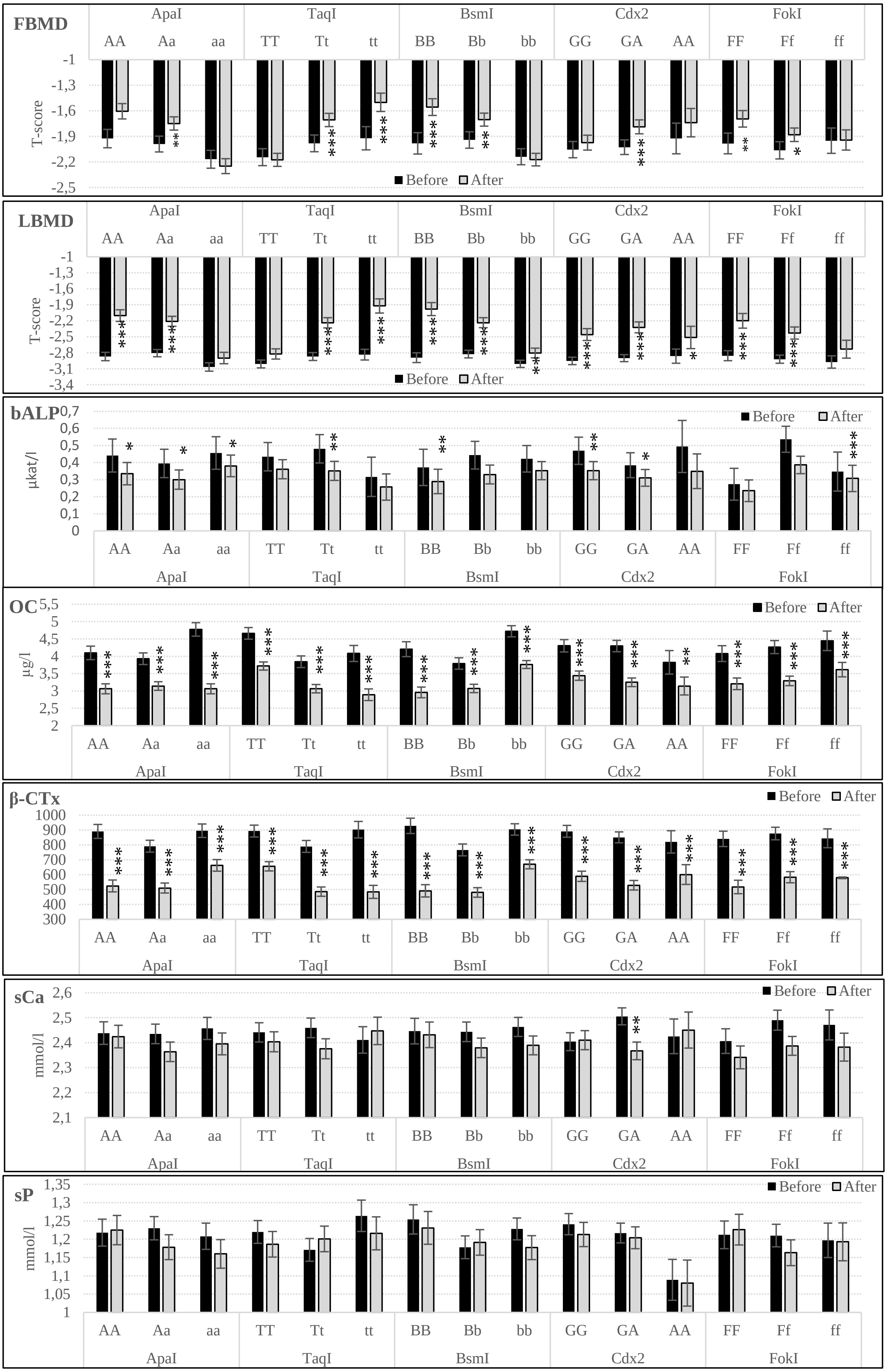

Figure S2: Effects of ibandronate therapy on osteoporosis-related traits across *VDR* genotypes. FBMD - femoral neck BMD, LBMD - lumbar spine BMD, bALP – bone isoenzyme of alkaline phosphatase, OC – osteocalcin, β-CTx - BetaCrosslaps, sCa - serum calcium, sP - serum phosphate, P values determine significant differences (***P<0.001; **P<0.01, *P<0.05).

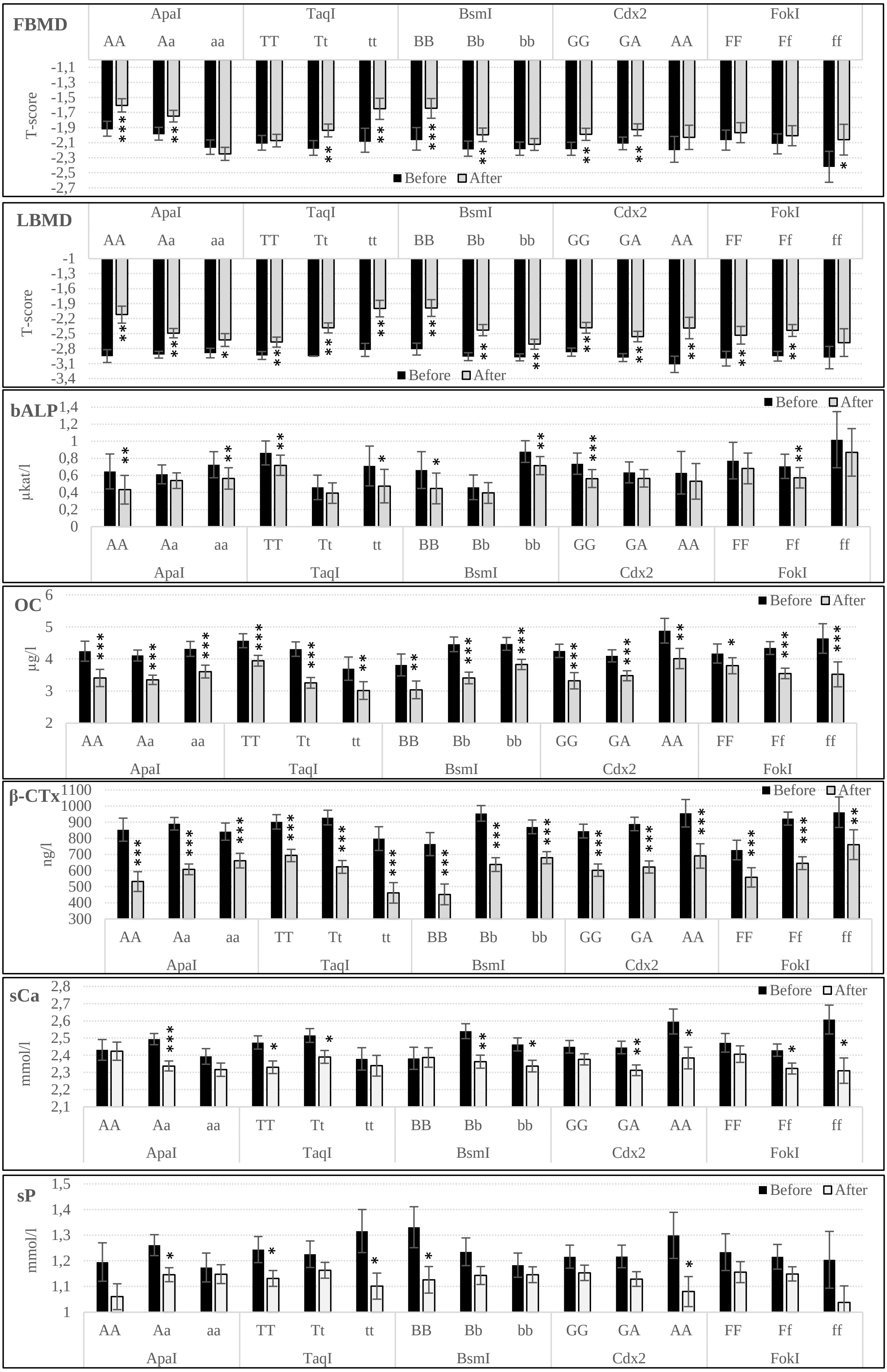

Figure S3: Effects of raloxifene therapy on osteoporosis-related traits across *VDR* genotypes. FBMD - femoral neck BMD, LBMD - lumbar spine BMD, bALP – bone isoenzyme of alkaline phosphatase, OC – osteocalcin, β-CTx - BetaCrosslaps, sCa - serum calcium, sP - serum phosphate, P values determine significant differences (***P<0.001; **P<0.01, *P<0.05).
